# Automated Extraction Improves Multiplex Molecular Detection of Infection in Septic Patients

**DOI:** 10.1371/journal.pone.0013387

**Published:** 2010-10-13

**Authors:** Benito J. Regueiro, Eduardo Varela-Ledo, Lucia Martinez-Lamas, Javier Rodriguez-Calviño, Antonio Aguilera, Antonio Santos, Antonio Gomez-Tato, Julian Alvarez-Escudero

**Affiliations:** 1 Clinical Microbiology Deparment, Complejo Hospitalario Universitario de Santiago, Santiago de Compostela, Spain; 2 Intensive Care Deparment, Hospital de Conxo, Complejo Hospitalario Universitario de Santiago, Santiago de Compostela, Spain; 3 School of Mathematics, Universidad de Santiago (Campus Sur), Santiago de Compostela, Spain; 4 Anesthesiology Deparment, Complejo Hospitalario Universitario de Santiago, Santiago de Compostela, Spain; Charité Universitätsmedizin Berlin, Germany

## Abstract

Sepsis is one of the leading causes of morbidity and mortality in hospitalized patients worldwide. Molecular technologies for rapid detection of microorganisms in patients with sepsis have only recently become available. LightCycler SeptiFast test M^grade^ (Roche Diagnostics GmbH) is a multiplex PCR analysis able to detect DNA of the 25 most frequent pathogens in bloodstream infections. The time and labor saved while avoiding excessive laboratory manipulation is the rationale for selecting the automated MagNA Pure compact nucleic acid isolation kit-I (Roche Applied Science, GmbH) as an alternative to conventional SeptiFast extraction. For the purposes of this study, we evaluate extraction in order to demonstrate the feasibility of automation. Finally, a prospective observational study was done using 106 clinical samples obtained from 76 patients in our ICU. Both extraction methods were used in parallel to test the samples. When molecular detection test results using both manual and automated extraction were compared with the data from blood cultures obtained at the same time, the results show that SeptiFast with the alternative MagNA Pure compact extraction not only shortens the complete workflow to 3.57 hrs., but also increases sensitivity of the molecular assay for detecting infection as defined by positive blood culture confirmation.

## Introduction

Sepsis is a clinical condition that is defined by objective signs establishing systemic inflammatory response syndrome (SIRS) together with the physician's suspicion of infection. Frequently, clinicians have to initiate early therapy with broad-spectrum empiric antibiotics before support is available from microbiological data that would confirm and focus their decisions [1]. Of the different strategies for treating the septic condition that ranges from systemic inflammatory response syndrome (SIRS) to septic shock and multiorganic failure, appropriate antibiotic therapy and prompt initial treatment have the greatest impact on reducing mortality and morbidity associated with sepsis [2], [3], [4]. In fact, there have been reports of an excessive in-hospital mortality of 31.4% in patients initially treated inadequately and who developed nosocomial infection in the ICU [5].

Recent publications [6], [7], [8] on the utility of molecular methods stress the potential advantages, in terms of aiding adequate therapy choice, of having a system that is able to rapidly diagnose and confirm an infection associated with SIRS. LightCycler SeptiFast test M^grade^ (Roche Diagnostics GmbH) is a multiplex real-time PCR based assay which is able, in about six hours, to detect the presence of DNA from 25 important bacterial and fungal species relevant for sepsis and nosocomial infection. The microorganisms covered by the test are the cause of approximately 90% of all blood stream infections and the range of species tested by SeptiFast includes those most frequently treated inadequately, as well as those producing the most difficult to treat infections [9], [10]. Like most diagnostic molecular tests, this method has a workflow designed in three phases: extraction, amplification/detection (real-time PCR) and data analysis. The nucleic acid isolation in SeptiFast test protocol includes successive steps of mechanical/enzymatic lysis and glass fiber filtration and is a mostly hands-on procedure for extraction that takes 3.52 hrs. The aim of the present study was to compare the efficacy of conventional extraction following SeptiFast manufacturer's recommendations against MagNA pure compact nucleic acid isolation kit I (cat. 03 730 964 001) (Roche Applied Science, Penzberg, GmbH), a rapid automatic DNA extraction method (approximately 34 min.) based on magnetic nanoparticles.

Our data show that automated MagNA pure compact extraction followed by SeptiFast detection not only shortens the complete workflow of the test from 6.54 hrs. to 3.57 hrs., but also increases assay sensitivity of the molecular assay for detecting infection defined by positive blood culture confirmation.

## Methods

### Extraction reagents controls

We used two types of DNA as controls for testing the extraction methods analyzed. Firstly, we used genomic Deoxyribonucleic acid from *Escherichia coli* Strain B (Sigma-Aldrich product num.: D4889 (USA)) as bacterial DNA standard for testing extraction. This DNA has been purified by equilibrium buoyant density gradient ultracentrifugation in cesium chloride, the solution was adjusted to a concentration of 5.30 A260 units of DNA per ml and lyophilized. Approximate average size is 16 kb and ratio A268/A280 is 1.9. *Escherichia coli* DNA is one of the targets included in SeptiFast test Master list.

And secondly, to evaluate the functionality of both methods we also used 20 reference microbial strains obtained from the American Type Culture Collection (ATCC) (USA): *Acinetobacter baumannii* (ATCC 19686), *Enterobacter cloacae* (ATCC 13847), *Serratia marcenscens* (ATCC 14756), *Stenotrophomonas maltophilia* (ATCC 51331), *Escherichia coli* (ATCC 25922) *Klebsiella pneumoniae* (ATCC 70063), *Proteus mirabilis* (ATCC 12453), *Pseudomonas aeruginosa* (ATCC 27853), *Staphylococcus aureus* (ATCC 25923), *Staphylococcus epidermidis* (ATCC 12228), *Streptococcus pneumoniae* (ATCC 49619) *Streptococcus agalactiae* (ATCC 13813) *Enterococcus faecalis* (ATCC 29212) *Enterococcus faecium* (ATCC 35667), *Candida albicans* (ATCC 90028) *Candida tropicalis* (ATCC 750) *Candida parapsilosis* (ATCC 22019), *Candida glabrata* (ATCC 1526), *Candida krusei* (ATCC 6258), *Aspergillus fumigatus* (ATCC 36607). The microbial sample mixture was composed of 10^4^ CFU/ml. of each strain in donor whole blood and genomic DNA from those strains was analyzed after extraction.

### Quality control

Two types of experiments were conducted to validate and control quality of extraction procedures performed:

(a) Fluorometric determination of DNA recovery as a measure of extraction yield. For DNA quantification we used the Quant-iT™ Assay Kit (Invitrogen, USA) with the Qubit® fluorometer following manufacturer instructions. The kit includes concentrated assay reagent, dilution buffer, and pre-diluted DNA standards. The assay is accurate for initial sample concentrations between 50 pg/µL and 200 ng/µL. Common contaminants, such as salts, free nucleotides, solvents, detergents, or protein are well tolerated in the assay.

In our study, serial dilutions (1∶2) of the E. coli DNA standard resuspended in TE buffer was extracted with the two extraction methods tested; recovery with MagNA pure compact was done using Total NA plasma 100–400 protocol that produces a final elution volume of 100 µl. Conventional SeptiFast manual extraction produces a final elution volume of 300 µl. Both extraction procedures were done and detection of strains in the mixture were performed by using SeptiFast® identification software, following manufacturer instructions.

(b). Testing of the performance of microbial SeptiFast® detection ability after extraction of reference strains using both methods. 20 reference strains obtained from American Culture Collection were mixed with a concentration of 10^4^ CFP/ml for each strain. Using equal amounts of each microorganism in donor whole blood. Genomic DNA from those strains was analyzed after extraction using both procedures here described. Melting temperature (Tm) and peak height for each strain was calculated after using SeptiFast® amplification, also adequacy of the values obtained was ascertained following the manufacturer criteria for assigning genera and species identification.

### Patient inclusion criteria and selection

A total of 72 patients from our Intensive Care and Anesthesiology Services, between May 2007 and May 2008, were included in the present study; all met criteria for SIRS and suspected sepsis on admission. Our study received hospital institutional review board approval (Comite de Investigacion, Complejo Universitario de Santiago) and patients' written informed consent was obtained before initiation at the intensive care unit. The final mortality rate of the group was 32.8%. Based on clinicians' suspicion of sepsis, 106 whole blood samples were obtained, consisting of one sample for SeptiFast and two bottles (aerobic and anaerobic) for blood culture (BacT-Alert (Biomerieux) France). When considered appropriate, additional blood culture series and other microbiology cultures from distinct compartments were taken for standard infection follow up. On the same days, biochemical and immunological techniques were performed to analyze additional clinical parameters. Clinical scores were done in all cases studied. The resulting clinical profile of the study group is shown in Table 1.

**Table 1:** Clinical Observational study summary.

**Patients num.**	72
**Samples num**	106
**Age**	64 (21–92)
**gender**	
male	53
female	19
**Mortality%**	37,5
**Basal Disease**	
respiratory	18
cardiovascular	18
alcoholism	10
oncologic	6
digestive	6
psychiatric	4
neurologic	2
various causes	6
other cause	2
**APACHE II**	
0–4	0
5–9	2
10–14	9
15–19	15
20–24	12
25–29	15
30–34	11
>34	8

Patients from our Intensive Care and Anesthesiology departments were sequentially selected between May 2007 and May 2008 to be included in the study, and had to meet criteria for SIRS and suspected sepsis on admission. Previous antibiotic treatment was not a criterion for rejection.

### Blood cultures and phenotypic Identification

Whole blood (10 ml) was collected for culture by either venous or arterial draw and inoculated in two resin containing blood culture bottles (FN/FA BacT-Alert, Biomerieux, France). Bottles were loaded into a BacT-Alert 3D automated blood culture instrument (Biomerieux, France). Once flagged by the instrument for detectable growth, fluid was withdrawn for gram stain and appropriate agar-based culture plates. Isolated colonies were analyzed either by an automated identification system (Vitek II (Biomerieux), France) or by appropriate biochemical phenotypic identification tests.

### Conventional microbiology

All other phenotypic or serological analysis processed to isolate microbial pathogens coming from body compartments other than blood are included under this heading. After gram and isolation in appropriate agar-based culture plates, selected colonies were analyzed and identified as above.

### SeptiFast® multiplex analysis

The LightCycler SeptiFast test M^grade^ is an in vitro nucleic amplification test for detection and identification of bacterial and fungal DNA from microorganism specified on the SeptiFast Master List and found in human K-EDTA blood using the LightCycler 2.0 Instrument (Roche Diagnostics GmbH). The test is used in conjunction with clinical presentation, established microbiological assays, and other laboratory markers to confirm bacterial/fungal blood stream infections.

The LightCycler SeptiFast test M^grade^ extraction is based on mechanical lysis of the 1,5 ml of whole blood specimens performed by using the SeptiFast Lysis kit and the Magna Lyser instrument (Roche Diagnostics GmbH). 1 ml. of lysed blood samples are incubated at elevated temperature with protease and chaotropic lysis buffer and, then, internal controls are added. Afterward, the mixture is transferred into a spin column with a glass fiber filter insert. The human genomic DNA and the microbial target DNA binds to the surface of the glass fiber, which is an unspecific absorption process. Unbound substances are removed in two consecutive washing steps. Then, the nucleic acid that is bound is eluted at elevated temperature and the eluates are subjected to PCR analysis. In our cases a dilution 1∶3 of the sample obtained is used for the next step. The real time PCR amplification of target DNA is done in three parallel reactions (gram positive bacteria, gram negative bacteria and fungi) and detection by specific Hybprobe probes is performed using the LightCycler 2.0 instrument (Roche Diagnostics GmbH) with uracil-N-glycosylase enzyme to prevent the risk of amplicon contamination. The emitted fluorescence is measured in one of the instrument's four different channels. After completion of amplification, a melting curve analysis is done and a report is generated by dedicated identification software (SeptiFast identification software). A detailed description of the test methodology has been described elsewhere [6].

### Alternative SeptiFast® protocol using MagNa pure extraction system

As with the conventional protocol, we start with mechanical lysis by using 1.5 ml of blood in one Septifast Lysis kit tube (cat. Num.:04 404 432 001) and shaking at 7000 rpm for 70 sc. on MagNA Lyser instrument (Roche Diagnostics GmbH). We use MagNA pure Compact Nucleic Acid Isolation Kit (cat. Num.: 03 730 964 001) according to manufacturer instructions. The protocol used for automated extraction is the Bacterial DNA protocol for the MagNA pure Compact instrument and we add 400 µl of blood lysed sample plus 4 µl of internal control for SeptiFast. The final elution volume is 200 µl. We follow manufacturer directions for the following SeptiFast amplification and detection steps.

Negative controls included in test do not work with MagNA pure instrument, so we use instead 400 µl of tissue-lysis buffer (4 M urea, 200 mM Tris, 20 mM NaCl,200 mM EDTA, pH 7.4) from high pure PCR template preparation kit (Roche Applied Science, cat. Num.:11-796-828-001, USA) plus 4 µl of internal control for SeptiFast® as an alternative for the negative control included in the commercial kit, which eventually may help to rule out possible contamination concerns. (For further information see “Data S1”)

### Statistical analysis

**Figure 1:**
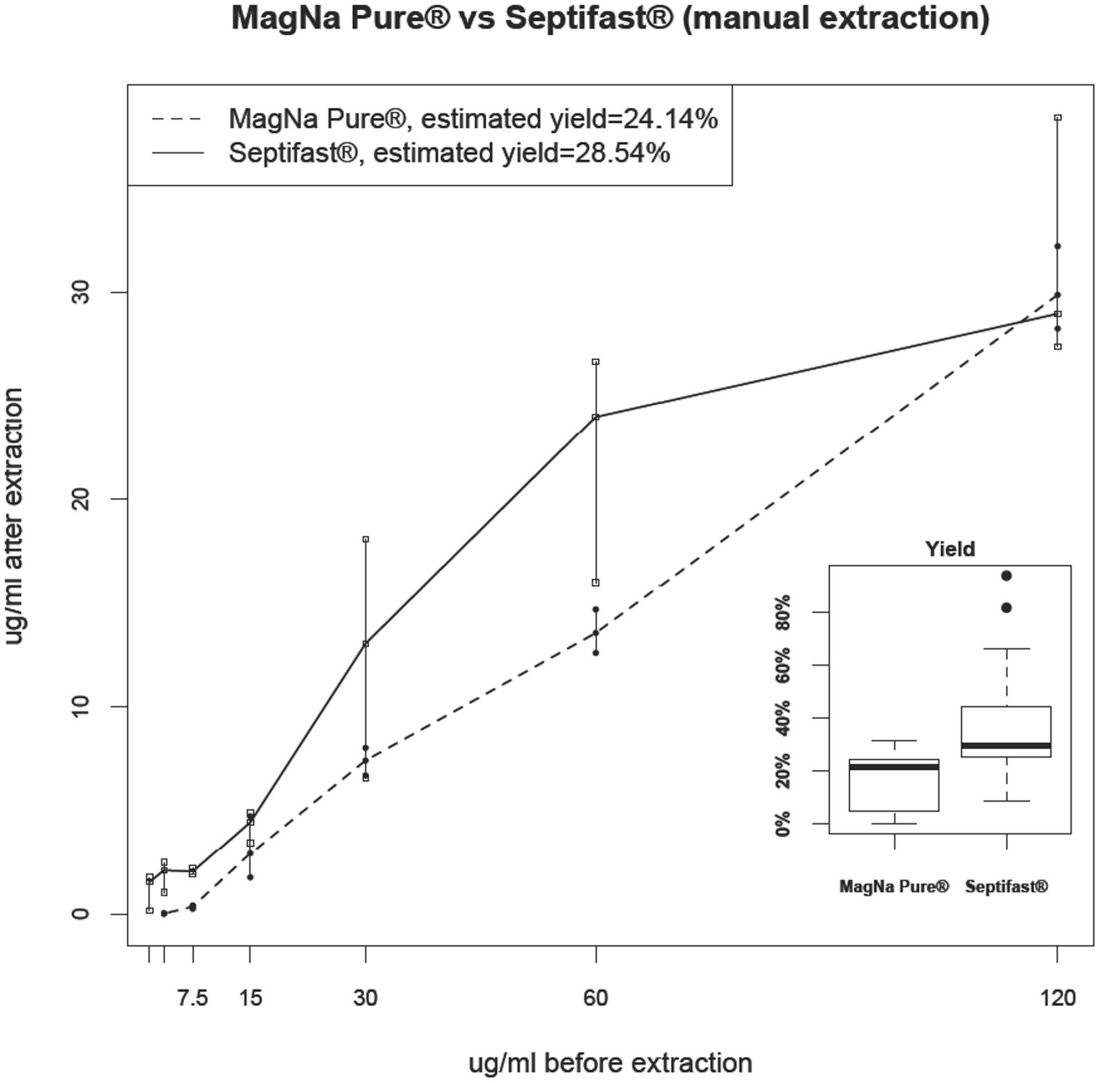
Comparative ability of the two extraction methods (SeptiFast® with manual extraction and SeptiFast® with MagNA pure compact extraction using protocol “Total NA Plasma 100–400”) using Escherichia coli DNA standard (Sigma Aldrich). SeptiFast® with manual extraction obtains more total amount of DNA than SeptiFast® with MagNA pure compact extraction. Points represent value of the media and also minor and maximum values for each dilution are represented. Boxplots represents the yields obtained using the two alternative methods, calculated as concentrations not as total product obtained in the eluate. (Wilcoxon rank sum test is W = 51, p-value = 3.89e-05).

The following linear model has been used to estimate yield: “*DNA recovered*” = “*yield*”×“*initial DNA quantity*”+“*error*”. Where “*error*” is assumed to be normally distributed with a mean of zero and a variance specified for each method. The estimated yield for each method is shown in the legend for Figure 1. Sensitivity, specificity and kappa analysis were performed using categorical variables in two-by-two tables (Data S2). Interpretation of strength of agreement for kappa was based on Landis R.J. and Koch G.G. criteria [11]. Analysis was performed by using R project for statistical computing.

## Results

### 1. Yield and DNA size of eluted samples after extraction using Septifast® with manual extraction vs. SeptiFast with MagNA pure compact extraction

We used E. coli standard DNA (Sigma-Aldrich (USA)) to test the comparative DNA recovery ability of the two extraction methods (SeptiFast with manual extraction vs. SeptiFast with MagNA pure compact extraction using protocol “Total NA Plasma 100–400”). The rationale for using this bacterial DNA standard is that the nucleic acid contained represents one of the microorganism included in SeptiFast master list. In fact, has additional functional and experimental advantages: it can be tested with the molecular SeptiFast detection system and renders positive.

To analyze extraction yield, we used the concentration of DNA recovered in the eluate after serial dilution to compare the ability of MagNA pure compact magnetic nanoparticles and the conventional technique with respect to unspecific absorption of nucleic acids. Up to the absorption limit of nanoparticles, we found the absorption capacity of both surfaces in terms of yields to be similar (24.14% for MagNA pure versus 28.54% for SeptiFast conventional extraction). Variability as well as and the total amount of blood processed (1000 µl vs. 400 µl) and the real volumes of final eluates (300 µl vs. 200 µl) were all higher using manual SeptiFast® extraction method. Data is shown in Figure 1.

### 2. Master list Strain performance on SeptiFast® detection with manual and automatic extraction

SeptiFast software employing a specific range of Temperature Melting (Tm) and peak height values was used to determine microbial species included on the master list and test the ability of both methods to recover microbial DNAs. After testing a mixture of 20 microbial strains (see Table 2), no discrepancies were found in the species reported by the instrument using both manual and automated SeptiFast methods.

**Table 2:** Tm and peak height values obtained for 20 different pathogens included in SeptiFast® test master list.

Assay	Microorganism	ATCC num.	Tm MP	Peak	TM SFM	Peak	SeptiFast® MP	SeptiFast® SFM
Gram negatives	*A. baumannii*	19686	65,38	0,42	64,00	0,08	+	+
	*E. cloacae*	13847	66,39	0,78	66,23	0,30	+	+
	*S. marcescens*	14756	58,00	0,36	58,00	0,24	+	+
	*S. maltophilia*	51331	63,09	0,16	63,08	0,38	+	+
	*E. coli*	25922	51,00	0,64	51,00	0,60	+	+
	*K. pneumoniae*	70063	59,22	0,12	59,01	0,19	+	+
	*P. mirabilis*	12453	55,01	0,25	54,63	0,32	+	+
	*P. aeruginosa*	27853	58,00	0,94	58,00	1,03	+	+
Gram positives	*S.aureus*	25923	61,81	0,12	61,42	0,09	+	+
	*S.epidermidis*	12228	51,60	0,08	51,43	0,11	+	+
	*S.pneumoniae*	49619	56,80	0,23	56,69	0,46	+	+
	*S.agalactiae*	13813	51,13	0,07	51,80	0,01	+	+
	*E.faecalis*	29212	62,78	0,03	62,29	0,02	+	+
	*E.faecium*	35667	54,61	0,06	54,60	0,05	+	+
Funghi	*C.albicans*	90028	55,92	1,90	55,77	1,82	+	+
	*C.tropicalis*	750	59,00	0,09	59,00	0,06	+	+
	*C. parapsilosis*	22019	54,97	0,06	54,00	0,03	+	+
	*C. glabrata*	1526	59,70	0,40	59,99	0,34	+	+
	*C. Krusei*	6258	51,77	0,27	51,66	0,55	+	+
	*A. fumigatus*	36607	56,68	0,42	58,51	1,61	+	+

After SeptiFast analysis of the 20-microbial-target sample mix, we compared melting temperature (Tm) and peak heights for both extraction methods, SeptiFast® with MagNA pure compact extraction (MP), and SeptiFast® with manual extraction (SFM). In order to be considered valid, values had to be within accepted ranges of the SeptiFast identification software (SIS). Melting temperature (Tm) and peak heights are presented along with SIS interpretation of values obtained.

### 3. Diagnostic values of SeptiFast detection with manual and automatic extraction in clinical samples

We tested 106 samples obtained from 72 ICU patients using both SeptiFast with the manual extraction protocol and SeptiFast with an alternative automatic extraction using MagNA pure compact. Results are shown in Tables 3 and 4.

**Table 3:** Results obtained testing samples in parallel.

Case Num. (Ref.)	MagNapure/SeptiFast result	Manual/SeptiFast result	BloodCulture/Vitek II result	Observations: other bacteriological cultures
1.- (169)	CoNS	CoNS	CoNs	
2.- (185)	**E. coli**	**Neg**	**Neg**	
3.- (194d2)	E. faecalis	**Neg**	E. faecalis	Ascitic fluid (+) Ent. faecalis
4.- (195)	**S. pneumoniae**	**Neg**	**Neg**	Sputum (+) S.pneumoniae
5.- (204d2)	E.faecium	E. faecium	E. faecium	Ascitic fluid (+) E. faecium
6.- (210)	S. pneumoniae	**Neg**	S. pneumoniae	
7.- (211d1)	S. pneumoniae	**Neg**	S. pneumoniae	
8.- (220)	CoNS	CoNS	CoNS	Catheter culture (+) CoNS
9.- (JAE)	**Neg**	**Neg**	**B. fragilis**	Ascitic fluid (+) B. fragilis
10.(235d3)	CoNS	**Neg**	CoNS	
11.(236d1)	E.coli	E. coli	E.coli	
12.(236d2)	E.coli	E.coli	E.coli	
13.(242d2)	K. pneumoniae/oxytoca	K.pneumoniae/oxytoca	K. pneumoniae	
14.(242d3)	K. pneumoniae/oxytoca	K.pneumoniae/oxytoca	K. pneumoniae	
15.(244d1)	S. aureus	S. aureus	**S. bovis**	
16.(251d3)	CoNS	**Neg**	CoNS	
17.-(255)	S. pneumoniae	**Neg**	S. peumoniae	Bronquial aspirate(+) S. pneumoniae
18.-(263)	C. albicans	S. aureus	C. albicans	Catheter, surgical wound (+) C. albicans
19.-(265)	**Neg**	**K. pneumoniae**	**Neg**	
20.(265d2)	CoNS	CoNS	CoNS	
21.(271d1)	S. aureus	S. aureus	S.aureus	Synovial fluid, Bronquial aspirate (+) S. aureus
22.(271d2)	St. aureus	S. aureus	S. aureus	Bronquial aspirate (+) S. aureus
23.-(272)	St. aureus	**Neg**	S aureus	
24.(280d1)	**Neg**	**Neg**	**E. faecalis/MRSA**	
25.-(311)	S. marcencens	S. marcencens	S. marcencens	Catheter tip (+) S. marcencens.
26.-(313)	S. marcencens.	S. marcencens	S. marcencens	
27.-(327)	C. parapsilosis	C. parasilopsis/P. aeruginosa	C parapsilosis	Urine (+) C. parapsilosis/Bronquial aspirate(+) P. aeruginosa
28.-(329)	S. aureus	S.aureus	S.aureus	
29.-(343)	E. cloacae/**E. coli**	E. coli	E. cloacae	Bronquial aspirate/wound exhudate (+) E. cloacae
30.- (355)	P. aeruginosa	**Neg**	P aeruginosa	Bronquial aspirate (+) P. aeruginosa
Additional 76 samples were **Negative** by all three methods				

Data obtained after testing 106 samples by three alternative methods, Negative (Neg) results and discrepancies are in bold. (Abbreviators: Bacteroides fragilis (B. fragilis) and Streptococcus bovis (S. bovis) Coagulase negative Staphylococci (CoNS) and Meticillin-resistant Staphylococcus aureus (MRSA) are bacteria not previously cited in the manuscript.)

**Table 4:** Differential diagnostic value of manual and automatic procedure.

A	LightCycler® Septifast MagNA Pure® compact extraction vs. LightCycler® Septifast manual extraction	LightCycler® Septifast manual extraction vs. Blood culture+VitekII identification	LightCycler® Septifast MagNA Pure® compact extraction vs. Blood culture+Vitek II identification.
Sensitivity	88.88 (65–98)	51.85 (31–71)	88.88 (70–97)
Specificity	87.5 (78–93)	98.73 (93–99)	96.2 (87–98)
Positive predictive value	59.25 (38–72)	93.33 (68–99)	88.88 (70–98)
Negative predictive value	97.46 (91–99)	85.71 (76–92)	96.2 (89–99)
Prevalence	16.98	25.47	25.47
Likelihood ratio Positive test	7,11 (3.99–12.65)	40.96 (5.64–297.03)	23.38 (7.64–71.53)
Likelihood ratio Negative test	0.126 (0.034–0.47)	0.487 (0.329–0.72)	0.115 (0.039–0.33)
B			
Proportion of agreement (strength of agreement)	0.87 (moderate)	0.86 (moderate)	0.94 (almost perfect)
Bias Index	0.08	−0.11	0
Prevalence Index	−0.57	−0.6	−0.49
Kappa (8)	0.637	0.592	0.85
C			
volume tested	400µl/1000µl	1000 µl/20ml.	400 µl./20 ml.

Note: (95% Confidence interval calculated with binomial expansion).

(See Supplemental data). **A**.: comparison of identification values obtained after using Accuracy matrix for both SeptiFast methods and blood culture+identification using Vitek II system. (Test Characteristics: Sensitivity: how good the test is at detecting an microorganism, Specificity: how good the test is at identifying patients with negative values, Positive predictive value: how often a patient with a positive test in fact has the microorganism, Negative predictive value: how often a patient with a negative test in fact does not have the microorganism.) **B**: Agreement matrix using kappa, which corrects the proportion of agreement due to chance and **C**: volumes of sample used in different experiments as reference for the compartment analyzed.

Firstly, the sensitivity and specificity measures of SeptiFast with automated extraction were compared to those for SeptiFast with manual extraction, which was used as a reference for positive infection. Then, successive comparisons of both SeptiFast procedures were performed using positive blood cultures as criteria for positive infection. Concordance was accepted when results were identical insofar as defining the species of the pathogen detected.

## Discussion

Sepsis is an inflammatory state resulting from the systemic response to bacterial infection. Conventional blood cultures are used for routine etiological diagnosis of confirmed bacteremia in septic patients. These tests are based on the viability of the microorganisms invading the blood compartment and laboratory reporting takes more than 24 hrs [12]. In the clinical setting, the need to start therapy with empiric broad spectrum antibiotics immediately after establishing clinical suspicion of sepsis frequently compromises our ability to isolate viable microorganisms in blood. Moreover, the size of the blood compartment and the amount of viable bacteria may also affect detection of disease in SIRS patients with suspected sepsis. Thus, clinicians are often forced to make critical empirical antibiotic decisions without the aid of microbiologically confirmed data [13].

In the present study we evaluate a fast molecular alternative for diagnosing pathogenic microorganisms directly from blood samples. Although molecular detection of microbial DNA in blood is not the same as conventional cultures based on viability, both methods are valid for defining infection and can be useful in current medical practice for improving our ability to follow up septic patients [14]. Conventional blood cultures detect viable microorganisms and make it possible to perform resistance analysis in vivo. On the other hand, molecular testing is not affected by antibiotic usage or any other viability issue and allows us to detect the presence of microbial DNA under conditions that would be adverse for conventional culturing methods.

Molecular diagnosis procedures involve three consecutive steps: nucleic acid extraction, amplification (PCR) and, detection and data analysis (target identification, normally carried out with dedicated software). In recent years a number of automated nucleic acid extraction methods have become available for use in conjunction with PCR techniques. These technologies are designed to be faster and more labor efficient. These automated methods reduce human error, improve precision, obtain reproducible results and allow analysis of large number of samples [15].

The aim of the present study was to evaluate an automatic method suitable for use with SeptiFast and ascertain if automating the protocol maintains detection sensitivity and specificity. In addition to the advantages mentioned above, this method would avoid possible interferences from manipulation in different laboratories. We evaluated two aspects, first extraction which was determined in terms of yields and second functionality, which is the ability to identify targets included on the test master list.

SeptiFast (Roche Diagnostics GmbH) is a multiplex PCR on real time analysis for simultaneous detection of 25 bacterial and fungal DNAs in whole blood and has been authorized for clinical diagnostic use in Europe. It requires up to 6. 54±0.27 hrs. [8] for reporting, furthermore, SeptiFast test currently uses a manual extraction procedure based on mechanical/enzymatic lysis and glass fiber filtration that involves extensive time (up to 211. 4 min) and labor, There are currently a number of choices for automating this process [16], [17].

We have selected MagNA pure compact automated system for SeptiFast extraction because it provides a number of advantages. Firstly, MagNA pure magnetic nanoparticles are one of the fastest extraction methods commercially available [18] and extraction takes only 34 min. (15 min of labor depending on the number of samples processed). Secondly, the total capacity of MagNA pure compact (8 samples per run) is well suited to use with SeptiFast. This alternative to the standard method produces a total testing time of 3.58 hrs, including mechanical lysis (42 min) and detection (139 min).

Using E. coli DNA standard, our findings indicate that this automated absorption process yields similar amounts of DNA as the conventional manual method (24.14% for MagNA pure versus 28.54% for SeptiFast conventional extraction) up to the point where the binding surface of MagNA pure nanoparticles (which is smaller than the binding surface for glass fibers) becomes saturated. Due to the presence of leukocytes, the amount of DNA in blood samples for septic patients is often above this saturation value and, thus, the final total amount of DNA extracted with the manual method is approximately three times the amount of DNA eluted with MagNA pure extraction [19].

To determine the automated test's level of functionality, we validated the correct identification of different microbial strains included on the SeptiFast® target master list. After SeptiFast analysis of the 20-microbial-target sample mix, we compared melting temperature (Tm) and peak heights for both extraction methods. In order to be considered valid, values had to be within accepted ranges of the SeptiFast identification software. As Table 2 indicates, Tm and peak data for standard ATCC microbial targets included in the master list presented acceptable values and fell within approved ranges. The rationale that SeptiFast uses for establishing these semiqualitative ranges is based on studies of bacterial DNA in blood samples from healthy subjects and has been adjusted for carriage contamination and reservoir of bacterial DNA in human blood [20].

In sum, the results show that MagNA pure extraction followed by LightCycler SeptiFast detection was valid under the standard conditions tested and constitutes a plausible alternative to conventional manual extraction. Moreover, it provides considerably savings in terms of labor time (49 min.) and extraction time (34 min.).

To test the clinical utility of SeptiFast with MagNA pure extraction, an observational prospective study of 72 patients was carried out. A total of 106 clinical samples were obtained involving parallel SeptiFast testing with both extraction methods as well as simultaneous blood cultures (2 bottles: one in aerobic and the other in anaerobic conditions). Concordance of both extraction methods was statistically analyzed by comparing validity measures with binary diagnostic tests (sensitivity and specificity), likelihood ratios, and positive and negative predictive value. These measures quantify a test's ability to distinguish individuals with and without a certain disease. In our case, an objective definition of disease is difficult [21], but for the purposes of comparison we have based our criteria for confirming infection in the blood compartment either on the results of the SeptiFast manual test or blood culture. As can be seen in Table 3, when using positive SeptiFast with manual extraction values as criteria for infection, SeptiFast with automatic extraction presents a sensitivity of 51.85%. Discrepancies between manual and automatic procedures may be due to differences in the amount of blood used that lead to differences in enzymatic effectiveness during the lysis step. In addition, the presence of genomic DNA or other DNA content may affect the unspecific absorption of microbial DNA detected by SeptiFast [22]. Similar sensitivity (56%) is also found when the conventional SeptiFast test is compared with positive simultaneous blood cultures. Besides the potential interference on unspecific absorption, discrepancies may also derive from the effect of previous antibiotic treatment as well as the isolation of viable microorganisms not included on test master list. Surprisingly, when we compared simultaneous positive blood cultures and SeptiFast with automatic extraction, we found a sensitivity of 92%. In fact, kappa values indicated an almost perfect agreement between the species defined after identification of isolates obtained in blood cultures and SeptiFast with MagNA pure compact extraction.

For the 27 positive and 79 negative cases identified by SeptiFast with MagNA pure compact extraction, specificity and positive and negative predictive values were consistently high compared to conventional SeptiFast or blood culture results. It is in positive values, where the rare discrepancies appeared. In fact, SeptiFast with MagNA pure extraction closely resembles blood cultures, but presents discrepancies with the manual extraction test. In our study, the six discrepancies between SeptiFast with automatic extraction and blood cultures involved the growth of one bacteria by blood cultures not covered on the SeptiFast list (*Bacteroides fragilis*). Another case was a *Streptococcus bovis*, which could be explained by the fact that one procedure uses phenotypic criteria for species assignment (Vitek II) while the other uses genotypic criteria (Internal transcribed spacer (ITS) region sequence homology). Two other bacteria were isolated from blood cultures but were negative for SeptiFast® (one *Staphylococcus aureus* meticilin-resistant and one *Enterococcus faecalis*), these cases and two other bacteria detected by SeptiFast® with automatic extraction but with negative cultures (*Escherichia coli* and *Streptococcus pneumoniae*) were detected in patients who received empiric antibiotic therapy previous to their inclusion in the study. Regarding manual and automatic SeptiFast® protocols, discrepancies basically involved negative results for conventional extraction and positive results for extraction with MagNA pure, except in one case, where *Klebsiella pneumoniae* was detected with the manual method while sample was negative for automatic extraction and blood culture.

Occasional discrepancies between multiplex real time PCR and positive simultaneous blood cultures have previously been reported [23], [24], [25] and should be reasonably expected in five situations: 1) when patients are previously treated with antibiotics that partially cover the bacteria, yielding positive molecular results and negative cultures, 2) when microorganisms are not included on the test master list, 3) when there is a mixed infection of microorganisms detected in the same LightCycler 2.0 channel, due to the competitive characteristics of the amplification procedure (limited availability of dNTPs), 4) when different criteria for assigning species identification is used in phenotypic identification (mainly biochemical properties) and molecular diagnosis (genetic homologies) and, finally, 5) in very few cases we had observed that certain species with similar Tms may be misinterpreted by the identification software of the molecular method (p.e., *Klebsiella oxytoca* and *Enterobacter cloacae*). All these discrepancies may be meaningful for individual cases, but only rarely occur when molecular diagnostic tests are used routinely. In any case, due to these possibilities it is always advisable to rely on a combination of clinical data, conventional cultures and molecular tests for the best patient follow up.

Another issue to consider is the need for precise clinical indications regarding when to use the test [26], [27], [28]. It is clear that not all patients benefit equally from the use of molecular diagnostic approaches [29]. The utility of the test depends on the patient's clinical status because the blood sample must adequately reflect the compartment that is going to be analyzed. Nevertheless, transient bacteremia, biofilms, and pathologies with low bacterial levels can affect the test's detection ability (i.e endocarditis or neutropenic patients) [30]. Despite some limitations that require professional judgment, the use of molecular diagnostic techniques constitutes an overall improvement in terms of providing well-grounded support for clinical therapeutic decisions.

In conclusion, our study has shown that SeptiFast test with MagNA pure compact extraction reduces workflow from 6. 54 hrs. to 3.56 hrs. when compared to SeptiFast with the conventional manual extraction. The use of this alternative automated protocol does not affect sensitivity or specificity of the method and actually increases assay sensitivity for detecting infection defined by positive blood culture confirmation. In our experience, combined use of rapid molecular testing and conventional microbiology represents an advantage for the septic patient and improves clinical decisions for achieving adequate treatment.

## Supporting Information

Data S1 (0.25 MB PPT)

Data S2 (0.06 MB DOC)
