# Supplementary material for: Automated Extraction Improves Multiplex Molecular Detection of Infection in Septic Patients
Source: PLoS One. 2010 Oct 13;5(10):e13387. doi: 10.1371/journal.pone.0013387 (PMC2954180; doi:10.1371/journal.pone.0013387)
Supplement: Data S2 — (0.06 MB DOC) [file pone.0013387.s002.doc]

SUPPLEMENTAL DATA

**SeptiFast® MagNa pure vs. SeptiFast® Conventional extraction methods**

Agreement Observer (Kappa):

|  | Yes | No | Total |
| --- | --- | --- | --- |
| Yes | 16 | 11 | 27 |
| No | 2 | 77 | 79 |
| Total | 18 | 88 | 106 |

**Kappa**: 0.637

Proportion agreement:0.87

Bias Index:0.08

Prevalence Index:-0.57

Accuracy Matrix:

**Infection defined by**

**Positive SeptiFast® (Convencional extraction)**

|  | Yes | No | Total |
| --- | --- | --- | --- |
| Yes | 16 | 11 | 27 |
| No | 2 | 77 | 79 |
| Total | 18 | 88 | 106 |

**Positive Test**

**Negative Test Sensitivity: 88.88 (65-98)**

**Specificity: 87.5 (78-93)**

**Likelihood ratio Positive test: 7.11 (3.99-12.65)**

**Likelihood ratio Negative test: 0.126 (0.034-0.47)**

**Positive predictive value: 59.25 (38-727**

**Negative predictive value: 97.46 (91-99)**

**Prevalence (index): 16.98**

**SeptiFast® Conventional extraction vs** **Blood Culture (Bact Alert/Vitek II identification) (BioMerieux)) methods**

Agreement Observer (Kappa):

|  | Yes | No | Total |
| --- | --- | --- | --- |
| Yes | 14 | 1 | 15 |
| No | 13 | 78 | 91 |
| Total | 27 | 79 | 106 |

**Kappa**: 0.592

Proportion agreement: 0.86

Bias Index. -0.11

Prevalence Index: -0.6

Accuracy matrix:

**Infection defined by positive Bloodculture**

|  | Yes | No | Total |
| --- | --- | --- | --- |
| Yes | 14 | 1 | 15 |
| No | 13 | 78 | 91 |
| Total | 27 | 79 | 106 |

**Positive Test**

**Negative Test Sensitivity: 51.85 (31-71)**

**Specificity: 98.73 (93-99)**

**Likelihood ratio Positive test: 40.96(5.64-297.03)**

**Likelihood ratio Negative test: 0.487 (0.329-0.72)**

**Positive predictive value: 93.33 (68-99)**

**Negative predictive value:85.71 (76-92)**

**Prevalence (index):25.47**

**SeptiFast® MagNa pure vs. Blood Culture (Bact Alert/Vitek II identification) (BioMerieux)) methods**

Agreement Observer (Kappa):

|  | Yes | No | Total |
| --- | --- | --- | --- |
| Yes | 24 | 3 | 27 |
| No | 3 | 76 | 79 |
| Total | 27 | 79 | 106 |

**Kappa**: 0.85

Proportion agreement: 0.94

Bias Index: 0

Prevalence Index: -0.49

Accuracy Matrix:

**Infection defined by**

**Positive Bloodculture**

|  | Yes | No | Total |
| --- | --- | --- | --- |
| Yes | 24 | 3 | 27 |
| No | 3 | 76 | 79 |
| Total | 27 | 79 | 106 |

**Positive Test**

**Negative Test Sensitivity: 88.88 (70-97)**

**Specificity: 96.2 (89-99)**

**Likelihood ratio Positive test: 23.38 (7.64- 71.53)**

**Likelihood ratio Negative test: 0.115 (0.039-0.33)**

**Positive predictive value: 88.88 (70-98)**

**Negative predictive value: 96.2 (89-99)**

**Prevalence (index): 25.47**
