# Supplementary material for: Automated Extraction Improves Multiplex Molecular Detection of Infection in Septic Patients
Source: PLoS One. 2010 Oct 13;5(10):e13387. doi: 10.1371/journal.pone.0013387 (PMC2954180; doi:10.1371/journal.pone.0013387)
Supplement: Supplementary file 2 [file pone.0013387.s001.doc]

LightCycler® Septi*Fast* Test Workflow

SeptiFast (Manual extraction) protocol

1,5 ml

Septi*Fast* Lysis Kit

1 ml

300 µl Eluted sample

Total time: 6.54 hrs
“hands on”: 228.4 min.

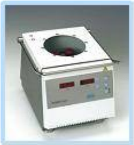

MagNa Lyser™
(Mechanical lysis)
- 32 min. sample preparation (7 samples)
- 11 min MagnaLyser and

Manual Extraction
185,4 min

MagnapureCompact Extraction
Bacterial Lysis (04 659 180 001)
40 min

Septi*Fast* Prep Kit

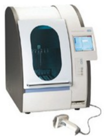

MAGNAPURE® compact

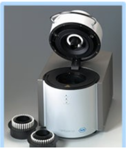

The LightCycler ®
Septi*Fast* Kit

LightCycler Amplification
-11 min sample preparation
- 127 min real-time PCR

Septi*Fast* Identification Software (SIS)

SeptiFast (Magnapure compact extraction) protocol

1,5 ml

Septi*Fast* Lysis Kit

0,4 ml+ 4 µl IC

200 µl Eluted sample

Total time: 3.68 hrs
“hands on”: 49 min.

Conditions:

-400 µl vs. 1 ml mechanically treated blood
-MagNa pure Compact extraction is not

USC
UNIVERSIDADE DE SANTIAGO DE COMPOSTELA
